# Genomic and Phenotypic Characterization of the Nontoxigenic Clostridioides difficile Strain CCUG37785 and Demonstration of Its Therapeutic Potential for the Prevention of C. difficile Infection

**DOI:** 10.1128/spectrum.01788-21

**Published:** 2022-03-22

**Authors:** Shaohui Wang, Joshua Heuler, Ishani Wickramage, Xingmin Sun

**Affiliations:** a Department of Molecular Medicine, Morsani College of Medicine, University of South Florida, Tampa, Florida, USA; University of California, Davis

**Keywords:** *Clostridioides difficile* infection (CDI), nontoxigenic *Clostridioides difficile* (NTCD), spores, therapeutics, microbial colonization, pathogenicity locus, genomics

## Abstract

Symptoms of Clostridioides difficile infection (CDI) are attributed largely to two toxins, TcdA and TcdB. About 17–23% of C. difficile isolates produce binary toxin, which enhances C. difficile pathogenesis. Previously, we engineered the nontoxigenic C. difficile strain CCUG37785 (designated as CCUG37785) to express immunogenic fragments of TcdA and TcdB as an oral mucosal CDI vaccine candidate. In this study, we performed genomic and phenotypic analyses of CCUG37785 and evaluated its potential use for preventing and treating CDI. Whole genome sequencing showed that CCUG37785 is ribotype ST3 and lacks toxin genes. Comparative analyses of PaLoc and CdtLoc loci of CCUG37785 revealed 115-bp and 68-bp conserved fragments in these regions, respectively. Phenotypic comparisons between CCUG37785 and C. difficile R20291 (an epidemic hypervirulent BI/NAPI/027 strain, designated as R20291) found that CCUG37785 exhibited significantly higher adhesion and sporulation, significantly lower spore germination and biofilm formation, and comparable motility to R20291. We also showed that oral inoculation of CCUG37785 spores prior to infection with R20291 spores provided mice almost full protection against developing CDI. However, oral inoculation of CCUG37785 spores after infection with R20291 spores only provided minor protection against CDI. Further analysis showed that mice pretreated with CCUG37785 spores secreted significantly less R20291 spores, while mice treated with CCUG37785 spores after infection with R20291 secreted a comparable amount of R20291 spores to mice infected with R20291 spores only. Our data both highlight the potential use of CCUG37785 for the prevention of primary and recurrent CDI in humans and support its use as an oral mucosal vaccine carrier against CDI.

**IMPORTANCE** Clostridioides difficile infection (CDI) symptoms range from diarrhea to intestinal inflammation/lesion and death and are mainly caused by two exotoxins, TcdA and TcdB. Active vaccination provides the attractive opportunity to prevent CDI and recurrence. No vaccine against CDI is currently licensed. Tremendous efforts have been devoted to developing vaccines targeting both toxins. However, ideally, vaccines should target both toxins and C. difficile cells/spores that transmit the disease and cause recurrence. Furthermore, C. difficile is an enteric pathogen, and mucosal/oral immunization would be particularly useful to protect the host against CDI considering that the gut is the main site of disease onset and progression. Data in our current study not only highlight the potential use of CCUG37785 to prevent primary and recurrent CDI in humans but also further support its use as an oral mucosal vaccine carrier against CDI.

## INTRODUCTION

Clostridioides difficile (C. difficile) is a Gram-positive, toxin-producing and spore-forming anaerobic bacterium (1). C. difficile is part of the normal intestinal microbiota in 1–3% of healthy adults, and 15–20% infants (2), and it is the leading cause of nosocomial antibiotic-associated diarrhea in developed countries. Moreover, a continued rise in the incidence of severe C. difficile infection (CDI) has been observed worldwide. Different studies from North America and Europe suggested that approximately 20–27% of all CDI cases were community-associated (2, 3). C. difficile produces three protein toxins, including toxin A (TcdA), toxin B (TcdB), and binary toxin (CDT), the first two of which are the major virulence factors of C. difficile and are the major drivers of CDI symptoms. CDT exists in about 17–23% C. difficile strains (4) and is associated with the increased severity of CDI (5, 6). According to reports from the Centers for Disease Control and Prevention (CDC) in 2017, there were 223,900 cases in hospitalized patients and 12,800 deaths in the United States.

Genes encoding TcdA (*tcdA*) and TcdB (*tcdB*) of toxigenic *C. diﬃcile* strains are located within the pathogenicity locus (PaLoc) (7). In the nontoxigenic *C. difficile* (NTCD) strains, the PaLoc region is replaced by a highly conserved 115/75-bp non-coding region (8, 9).

NTCD strains have been explored as a safe and effective preventive for CDI (10–14). A hamster model was used to evaluate the effectiveness of a NTCD strain as a preventive agent for the infection with toxigenic *C. diﬃcile* (15), and the authors found that the prevention of CDI mortality was correlated closely with the NTCD colonization prior to challenge with toxigenic *C. diﬃcile* strains. In 2013, the same group found that NTCD colonization was displaced by the toxigenic strain (16) in some hamsters used in experiments, suggesting that toxigenic and NTCD strains may compete for colonization. A possible mechanism by which *C. diﬃcile* strains establish or lose colonization in the intestine is that strains differ in their ability to adhere to colonic mucosal cells or mucus (14, 17). Both adherence ability and motility are important for *C. diﬃcile* strains to establish colonization. CDI has very high rates of recurrence/relapse after treatment with antibiotics. NTCD strain administration after CDI treatment may serve as a preventive strategy against recurrent CDI (14, 18).

Previously, we engineered the nontoxigenic strain C. difficile CCUG37785 (designated as CCUG37785) to express mTcd138, which is comprised of the glucosyltransferase and cysteine proteinase domains of TcdB and the receptor binding domain of TcdA, generating the strain NTCD_Tcd138 as an oral mucosal vaccine candidate against CDI. Our data indicate that NTCD_Tcd138 is a promising oral vaccine candidate against CDI (19). It was the first report on the use of nontoxigenic C. difficile strains-based vaccines against CDI. In this study, we aimed to perform comparative genomic and phenotypic analyses of the strain CCUG37785 in comparison with other relevant C. difficile strains and to determine if CCUG37785 can protect mice against infection with a toxigenic C. difficile strain.

## RESULTS

### Genome analysis of C. difficile CCUG37785.

**FIG 1:**
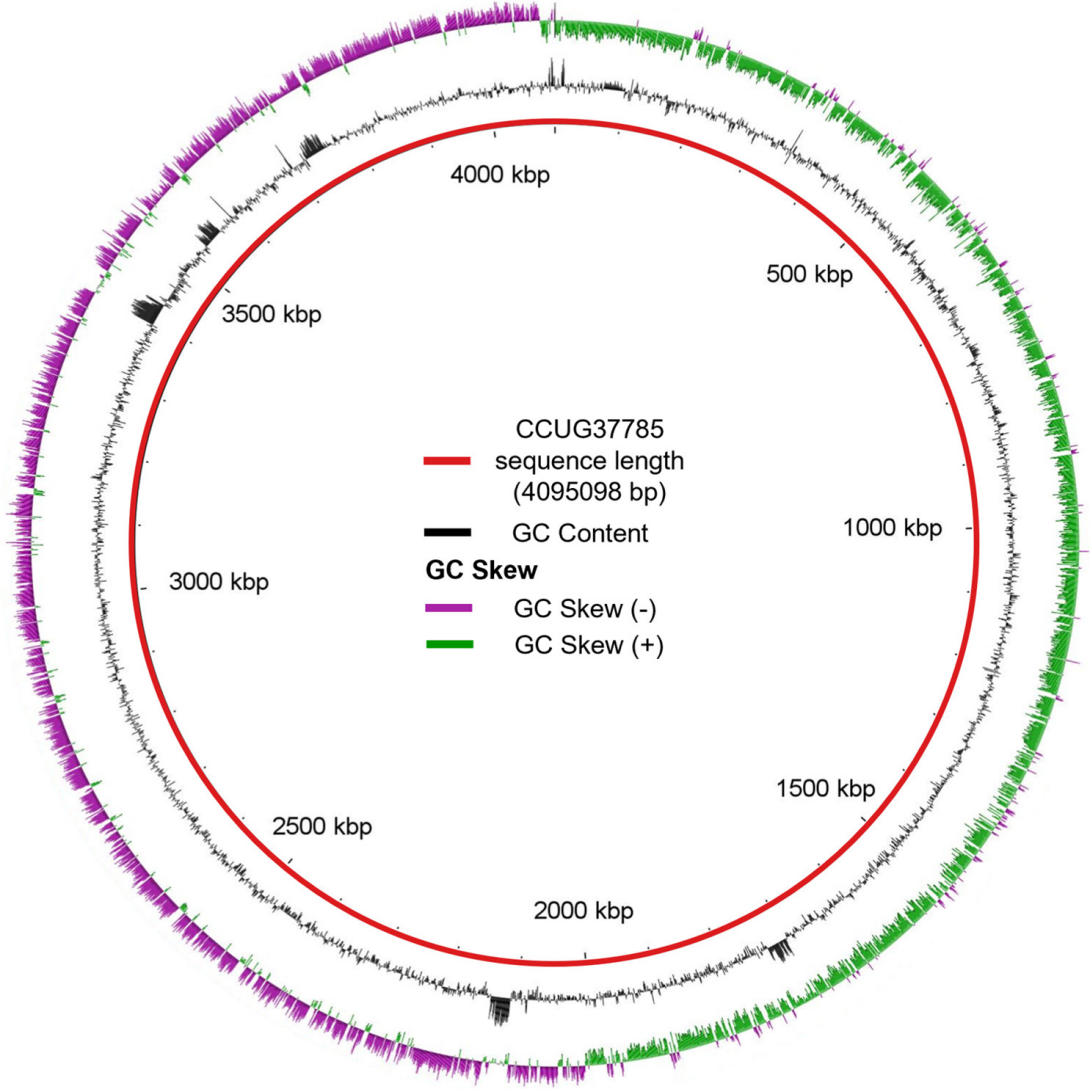
The GC content (black graph) and GC skew (green and purple graph) of the CCUG37785 genome. Figure was made with BLAST Ring Image Generator (BRIG).

**FIG 2:**
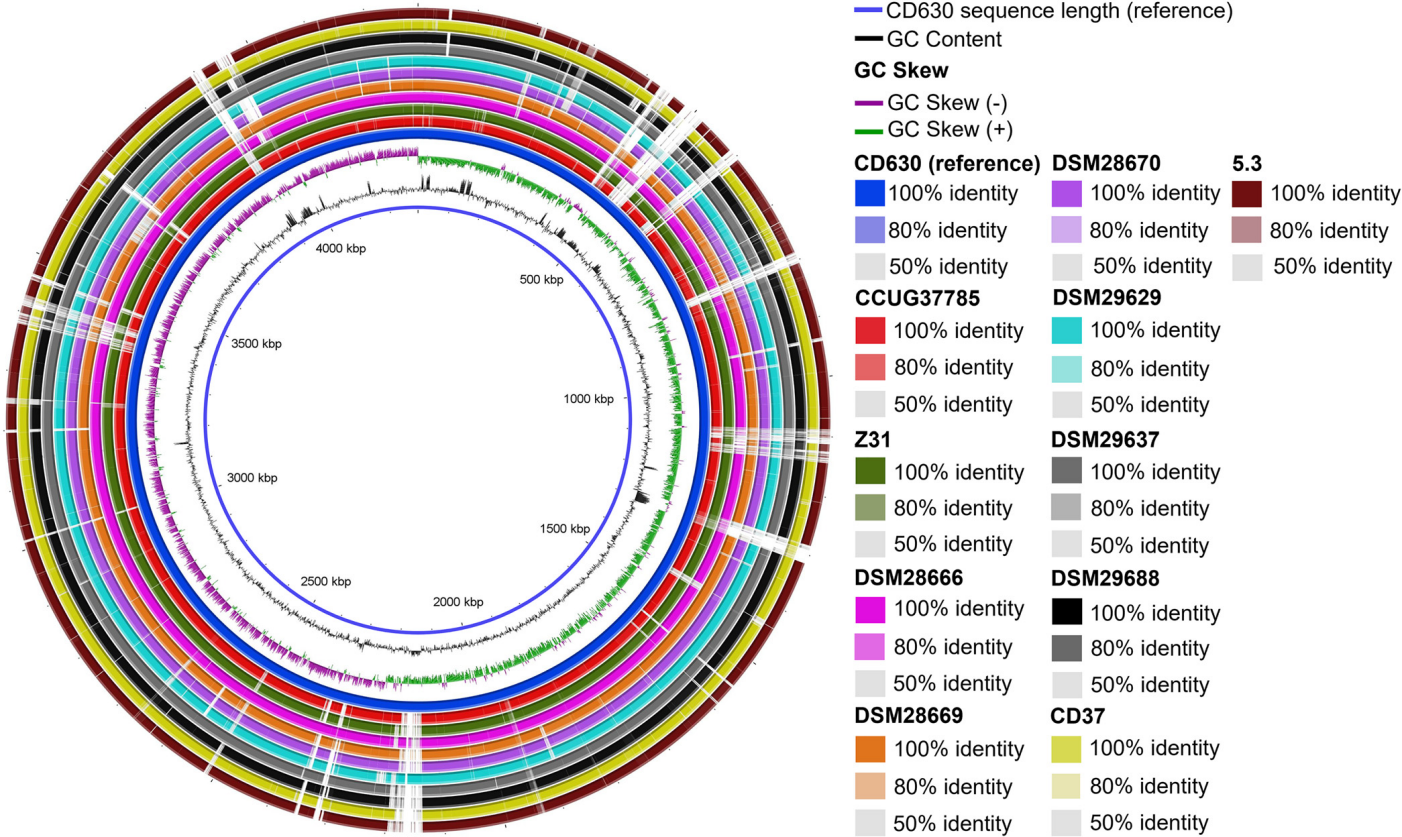
Sequence identity between nontoxigenic C. difficile strains. The colored rings illustrate shared sequence identity between the indicated nontoxigenic strains and CD630 (reference strain). White space within the rings indicates low (<50%) sequence identity between the given strain and CD630. The inner blue line indicates the length of the CD630 genome. The GC content and GC skew graphs of strain CD630 are also indicated. Figure made with BLAST Ring Image Generator (BRIG).

Whole genome sequencing, conducted at a sequencing depth of approximately 300X, yielded high quality reads, which were assembled into a draft genome with an N50 of 323,436 and a maximum contig length of 793,487. Strain CCUG37785 has a genome size of 4,095,098 bp, with a GC content of 28.8% in line with the 28–30% GC content typically observed in C. difficile genomes (Table 1). The GC content and skew graph of CCUG37785 (Fig. 1) are characterized by a high-GC content region (0–2 Mbp) followed by a relatively low-GC content region spanning the remainder of the genome, which is similar to those of CD630 (Fig. 2).

**TABLE 1:** Sequenced nontoxigenic C. difficile strains in the literature^*a*^

Name	Size (bp)	Plasmids	GC%	CDS	tRNA	rRNA	Accession	Type	Reference
CCUG37785	4,095,098	0	28.8	3733	48	5	JAGKRT010000000	WGS	This study
CD37	4010233	0	28.6	3,695	16	5	AHJJ00000000.1	WGS	21
Z31	4298263	0	29.2	4,128	58	29	CP013196	CG	22
5.3	4009318	0	28.3	3,580	82	46	AZSH00000000.1	WGS	14
DSM 28666	4122585	1	29.0	3802	89	36	CP012321.1	CG	24
DSM 29637	4114585	0	28.6	3833	89	35	CP016106.1	CG	
DSM 29688	4228964	0	28.9	4022	90	35	CP019858.1	CG	
DSM28670	4191653	0	28.8	3972	91	35	CP012312	CG	
DSM 29629	4111235	0	28.6	3799	90	35	CP016104	CG	
DSM 28669	4137693	0	28.8	3901	90	35	CP012323	CG	

a GenBank annotations of nontoxigenic strains were downloaded as either whole-genome shotgun (WGS) sequences or as complete genome (CG) files for use in further analysis.

The sequence type (ST) of this isolate based on public MLST typing was determined to be ST3. The RAST annotation software predicted 48 tRNA genes and 5 rRNA genes in CCUG37785 (Table 1). No plasmids were predicted in CCUG37785 after searching with PlasmidFinder Software (20). Compared with the reference genome of toxigenic strain CD630, the CCUG37785 genome contained none of the toxin genes of the PaLoc (*tcdA*, *tcdB*, *tcdR*, *tcdC or tcdE*) or CdtLoc (*cdtA* or *cdtB*). The result was confirmed by our toxin expression assay by ELISA (Fig. S1 in the supplemental material) and PCR (data not shown). For comparison, sequenced nontoxigenic C. difficile strains were mined from the literature (21–24) (Table 1). Strain CD37, for example, was the first nontoxigenic C. difficile with draft genome analyzed (21), whereas strain Z31 was the first nontoxigenic C. difficile strain with its complete genome sequence reported and studied (22).

**FIG 3:**
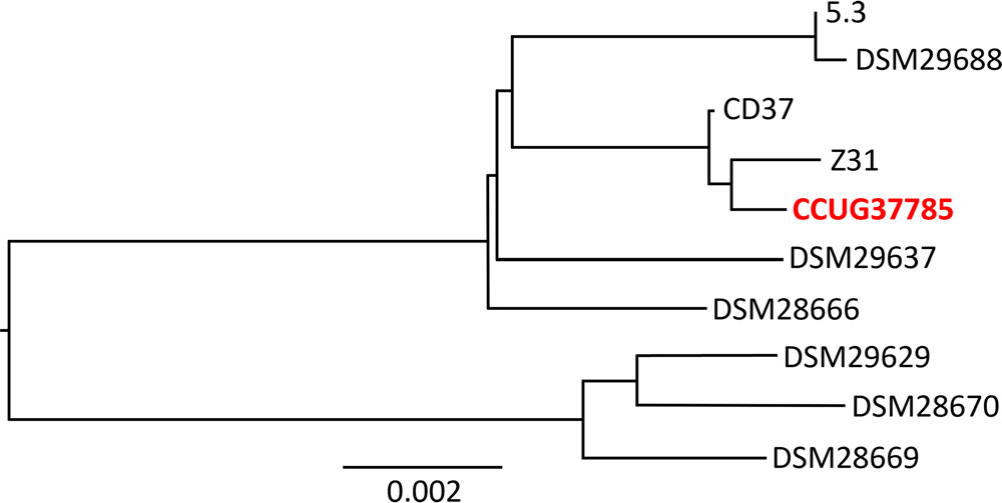
Phylogenetic tree of nontoxigenic C. difficile strains. A minimum evolution phylogenetic tree was generated using the Type (Strain) Genome Server (TYGS).

Genomes of all 10 strains in Table 1 were uploaded to the Type Strain Genome Server (TYGS) (25) to generate a minimum evolution phylogenetic tree (Fig. 3). Strain CCUG37785 is most closely related to strains Z31 and CD37. Yet, the scale line in Fig. 3 indicating 0.002 nucleotide substitutions per site suggests that, in fact, all the nontoxigenic strains included in the phylogenetic tree share significant sequence identity with CCUG37785. BLASTn analysis confirms this observation, as using the CCUG37785 genome as a query to search against nontoxigenic strains reveals that other strains share >98% identity and >90% query cover with CCUG37785 (Table S1 in the supplemental material). The selected nontoxigenic strains share significant sequence identity with the toxigenic reference strain CD630 (Fig. 2). Also, while nontoxigenic strains contain some genomic regions with low sequence identity to CD630 (<50%, white regions in each ring), these occur at similar locations in the nontoxigenic strains analyzed (e.g., ∼500 kbp, ∼800 kbp, etc.).

### Prophages.

Previously reported nontoxigenic C. difficile strains have been found to encode numerous prophage regions, such as those of Z31 (22). In CCUG37785, the PHASTER program predicted 3 incomplete prophage-like regions and one putative complete prophage (Table 2). The intact prophage (region 3) was predicted to have a length of 56.6 Kb with a GC content of 29.2%, which are both in agreement with the length and GC% ranges observed in C. difficile bacteriophages (26). A draft annotation of the intact prophage-like region is provided in Table S2 in the supplemental material.

**TABLE 2:** Putative prophage carriage in CCUG37785

Region	Length	Position	Completeness	Score	# Putative ORFs	%ORFs with an assigned function	GC %
1	13.7Kb	234102-247839	Incomplete	30	11	100	30.2
2	27.4Kb	1313979-1341418	Incomplete	60	31	67.7	27.6
3	56.6Kb	1554571-1611218	Intact	150	76	60.5	29.2
4	5.8Kb	2455294-2461108	Incomplete	20	19	57.9	23.8

### Comparative analysis of PaLoc.

The pathogenicity locus (PaLoc) of C. difficile is made up of a 19.6 kb region encoding five genes including the large clostridial toxins A and B (27), which are the main cause of the negative health effects associated with C. difficile infection (28–30). The other three surrounding genes are *tcdR*, *tcdC*, and *tcdE*. Gene *tcdR* plays a role in positive transcriptional regulation, *tcdC* negatively regulates toxin transcription, and *tcdE* facilitates toxin secretion (27, 31, 32). The PaLoc region is highly conserved, and nontoxigenic strains lack the entire region (33). Instead, nontoxigenic strains contain a conserved 115-bp sequence in place of the PaLoc region that is unique to nontoxigenic strains (8, 9, 27, 31, 34).

**FIG 4:**
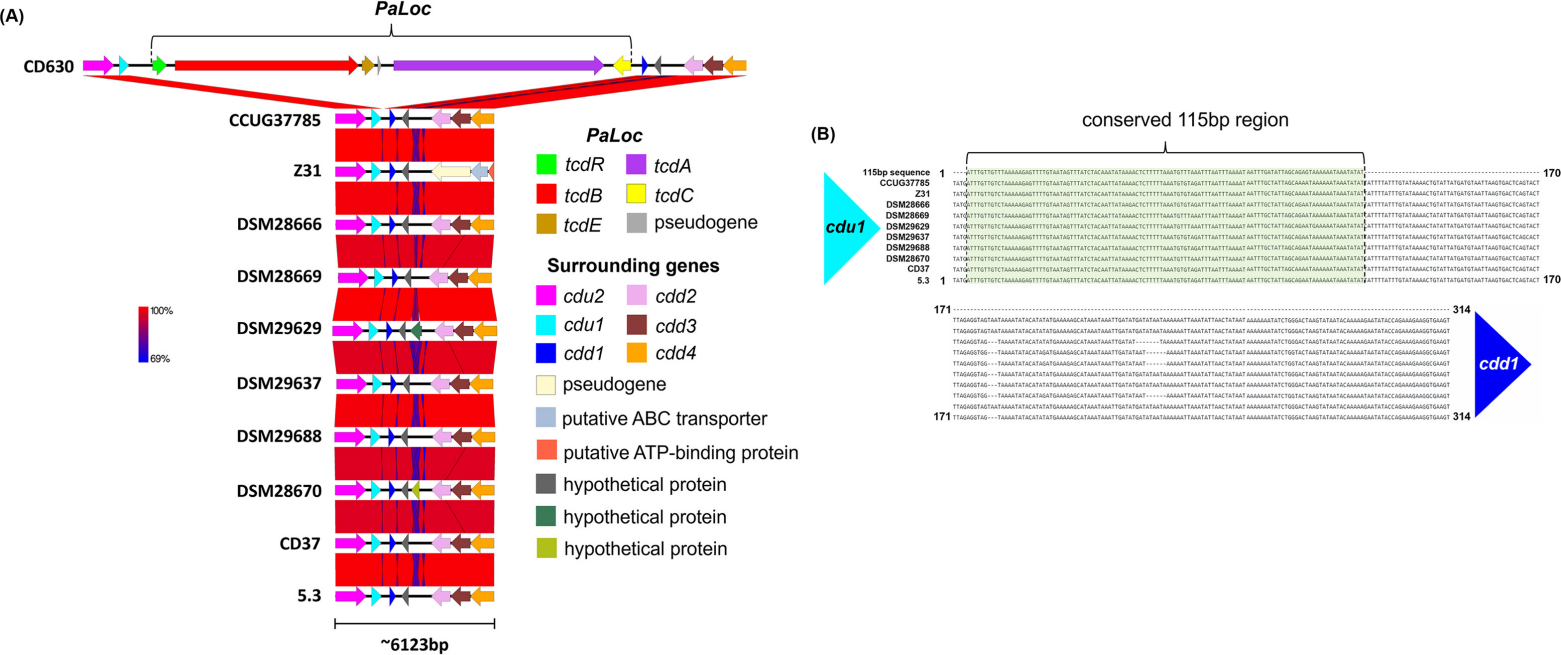
(A) Comparative analysis of PaLoc region. Downstream of *cdu2* (pink) and *cdu1* (light blue), toxigenic strains like CD630 encode toxin-related genes starting with *tcdR* (green) and ending with *tcdC* (yellow). Downstream of the PaLoc, CD630 encodes *cdd1* (dark blue), *cdd2* (pink), *cdd3* (maroon), and *cdd4* (orange). Nontoxigenic strains share virtually 100% identity with these aforementioned genes upstream and downstream of the PaLoc region, but nontoxigenic strains substitute the toxin-related genes for a 115 bp insert in between *cdu1* and *cdd1*. (B) Conserved sequences replace the PaLoc in nontoxigenic strains. Between the *cdu1* and *cdd1* genes, nontoxigenic strains encode a conserved 115 bp sequence in place of the typical PaLoc genes of toxigenic strains. Sequences were mined from their respective genomes and aligned using MAFFT.

Upstream of the PaLoc region (or 115-bp insert in nontoxigenic strains), C. difficile genomes encode the genes *cdu1*, *cdu2*, and *cdu3*, while downstream genes include *cdd1*, *cdd2*, *cdd3*, and *cdd4* (31). In light of this knowledge, we also mined the PaLoc and surrounding regions of CCUG37785 in comparison with those of CD630 and the sequenced NTCD strains listed in Table 1. Comparative analysis of PaLoc regions demonstrates how the arrangement of genes adjacent to PaLoc is conserved in toxigenic and nontoxigenic strains (Fig. 4A). We demonstrate that the *cdu1* and *cdu2* genes upstream of the PaLoc of strain CD630 share high sequence similarity to genes upstream of the PaLoc of CCUG37785 and other nontoxigenic strains. The downstream genes of the CD630 PaLoc also display high similarity with the sampled strains. In between the *cdu1* and *cdd1* genes, toxigenic strains would normally encode the toxin genes and regulatory genes of PaLoc, but CCUG37785 and other nontoxigenic strains have a conserved 115-bp sequence as reported (31) (Fig. 4B). CCUG37785 genome contains a 115-bp sequence upstream of *cdd1* (dark blue) and downstream of *cdu1* (light blue) that is identical to the reported sequence of the insert characterized in the reported nontoxigenic strains (Fig. 4B).

### Comparative analysis of CdtLoc.

Hypervirulent strains of C. difficile sometimes also encode a binary toxin (CDT), which is associated with greater disease severity (5). The CdtLocus (CdtLoc) is a 6.2-kB region that encodes the CDTb and CDTa toxins along with a regulatory gene *cdtR* (35, 36). While the PaLoc region can exist in many shortened forms depending on the specific strain (37), CdtLoc is either entirely present or absent in C. difficile genomes (38). C. difficile genomes that lack the CdtLoc regions instead have a conserved 68-bp sequence that is not found in binary toxin-positive strains (36). The CdtLoc or the 68-bp sequence is flanked upstream by CD2601 and CD2602 and downstream by *trpS*, the response regulator associated with *cdtR* (5).

**FIG 5:**
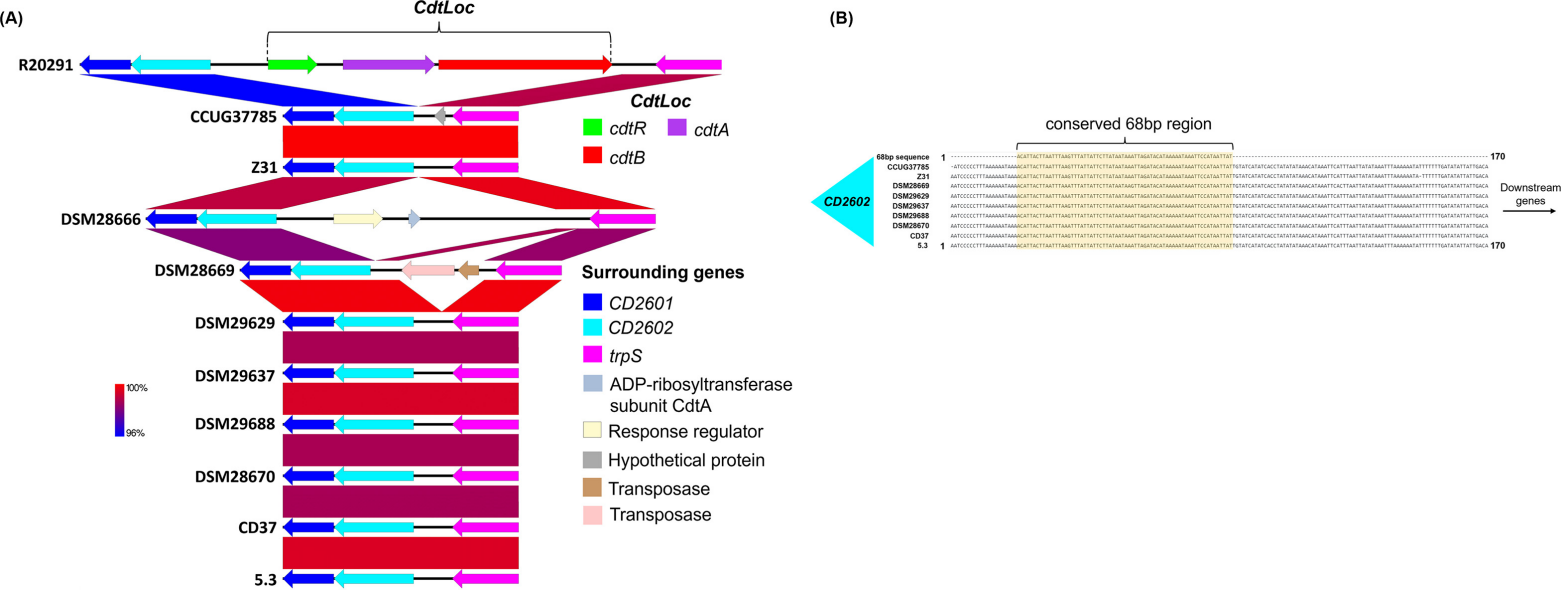
(A) Comparative analysis of CdtLoc region. The example binary toxin-positive strain CD196 encodes the genes *CD2601* (dark blue) and *CD2602* (light blue) upstream of binary toxin-related genes and *trpS* downstream. Nontoxigenic strains also encode *CD2601*, *CD2602*, and *trpS*, but the binary toxin genes are absent. DSM28666 and DSM28669 do, however, encode genes for a response regulator (cream), hypothetical proteins (gray), and transposases (pink, brown) that are not homologous to sequences at this genomic locus/in this region in other nontoxigenic strains. (B) Conserved sequences replace the CdtLoc in nontoxigenic strains. Between the *CD2602* and *trpS* genes, nontoxigenic strains encode a conserved 68 bp sequence in place of the typical CdtLoc genes of binary toxin-positive strains. Sequences were mined from their respective genomes and aligned using MAFFT.

Comparative analysis of CdtLoc regions shows that the genes upstream of CdtLoc including *CD2601* (dark blue) and *CD2602* (light blue) are present in nontoxigenic strains including CCUG37785 (Fig. 5A). *trpS*, which is encoded downstream of the CdtLoc in R20291, is also present in all the nontoxigenic strains examined. The CdtLoc appears to be more heterogeneous than the PaLoc regions shown in Fig. 4, as DSM28666 and DSM28669 display additional sequences and/or genes between CD2602 and *trpS* that are dissimilar to other strains. Further analysis of the sequences between *CD2602* and *trpS* is shown in Fig. 5B. The figure illustrates that most of the CDT-negative strains examined, including CCUG37785, contain the conserved 68-bp sequence in place of CdtLoc. DSM28666 is the one exception to this trend, as no significant similarity was found between the 68-bp sequence and the DSM28666 genome.

### Strain CCUG37785 shows significantly higher sporulation rates and adherence to human intestinal epithelial cells than R20291.

**FIG 6:**
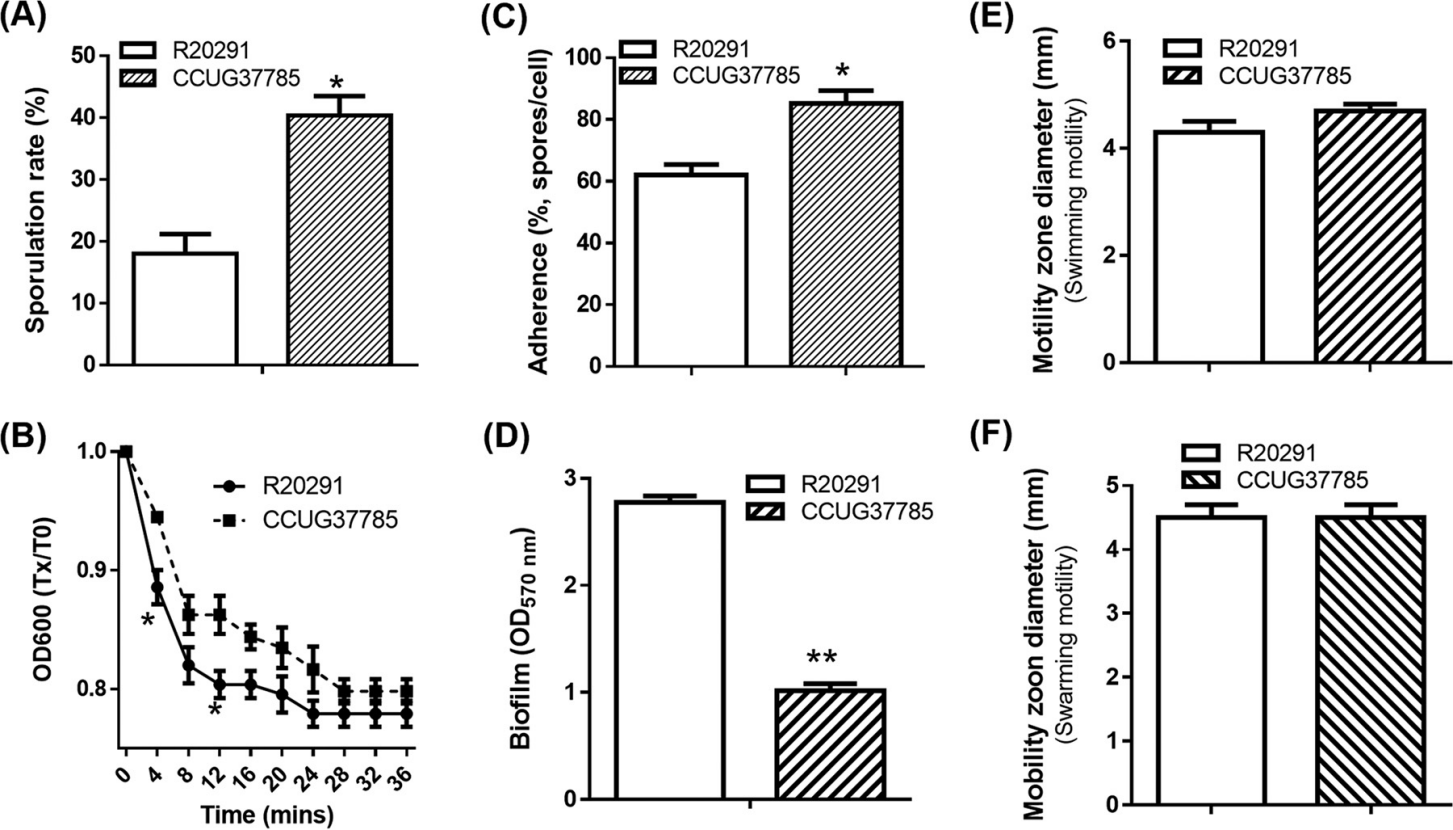
Comparison of C. difficile strains R20291 and CCUG37785 in sulation, germination, adhesion, motility, biofilm formation and motility. (A) Sporulation assay. (B) Germination assay. For C. difficile germination analysis, the purified spores were diluted to an OD_600_ 0f 1.0 in the germination buffer. The value of OD_600_ was monitored immediately (0 min, t_0_), and was detected once every 2 min (t_x_) for 20 min at 37°C. The germination ratio was calculated as OD_600_ (Tx)/OD_600_ (T_0_). Spores in germination buffer without TA was used as the negative control. (C) Adherence of C. difficile vegetative cells to HCT-8 cells *in vitro*. (D) Biofilm formation analysis. (E and F) Halo diameter of motility zones (swimming analysis on 0.175% agar plate, swarming analysis on 0.3% agar plate). Experiments were independently repeated thrice. One-way analysis of variance (ANOVA) was used for statistical significance. Data are present as “Mean±SD”. ∗, *P* < 0.05; ∗∗, *P* < 0.01.

We first compared strain CCUG37785 with R20291 in sporulation and adherence to intestinal epithelial cells. As shown in Fig. 6A, CCUG37785 exhibited a significantly higher frequency of sporulation (40.33 ± 5.38%), than R20291 (18.03 ± 4.73%; *P < 0.05). Purified *C. difficile* spores were used to evaluate their adherence to HCT-8 cells. CCUG37785 showed a significantly higher adherence rate (85.22 ± 4.15%) than R20291 (62.00 ± 3.47%) (Fig. 6C; *P < 0.05).

### Strain CCUG37785 exhibits lower germination rates and biofilm formation, but comparable motility in comparison with R20291.

The germination capability of CCUG37785 spores was evaluated. As shown in Fig. 6B, strain CCUG37785 exhibited significantly lower germination rates than R20291 (*P < 0.05). The capability of C. difficile CCUG37785 to form biofilms was also evaluated by crystal violet (CV) staining after growth in microtiter plates. Strain CCUG37785 exhibited significantly lower levels of biofilm formation than R20291 (Fig. 6D, **P < 0.01). To assess the motility of CCUG37785 vegetative cells, swimming and swarming assays were performed. As shown in Fig. 6E and F, strain CCUG37785 showed similar motility to strain R20291 (swimming assay, *P* = 0.1265; swarming assay, *P* < 0.999).

### Strain CCUG37785 protects mice against infection with an epidemic *C. difficile* strain.

**FIG 7:**
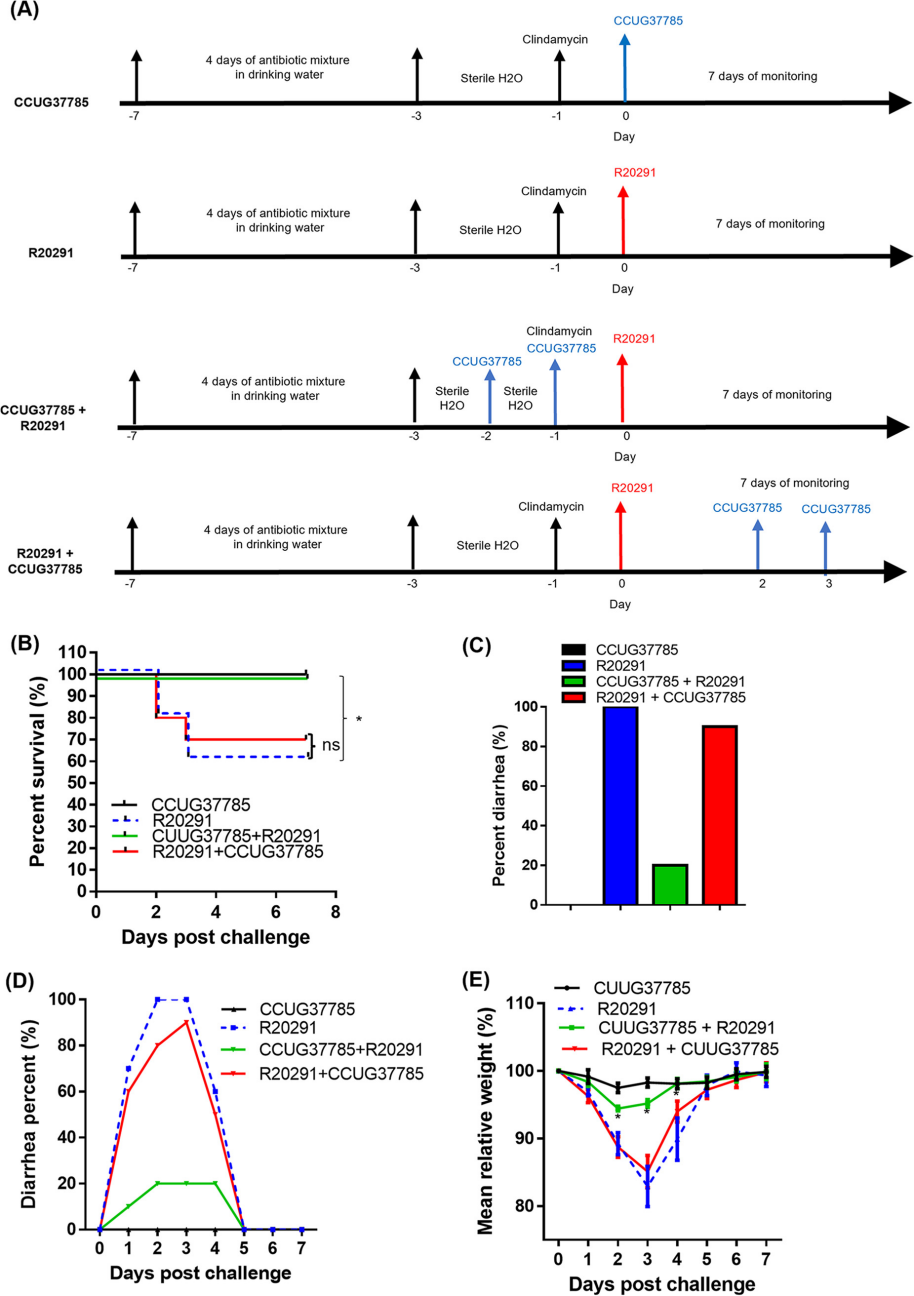
C. difficile CCUG37785 protects mice against infection with *C. difficile* R20291. (A) Experimental scheme of testing if strain CCUG37785 can protect mice against infection with the strain R20291. After 4 days of pretreatment with an antibiotic mixture, mice were given autoclaved water for 2 days, followed by a single dose of intraperitoneal injection with clindamycin (10 mg/kg) 1 day before (day-1) challenge with R20291 spores by gavage (day 0). Group 1 (CCUG37785) was challenged with 10^6^ CCUG37785 spores on day 0; Group 2 (R20291) was challenged with 10^6^ R20291 spores on day 0; Group 3 (CCUG37785/R20291) was given 10^6^ CCUG37785 spores by gavage on days -1 and -2, followed by challenge with 10^6^ R20291 spores on day 0; Group 4 (R20291/CCUG37785) was infected with 10^6^ R20291 spores on day 0, followed by giving 10^6^ CCUG37785 spores by gavage on days 2 and 3. Mice were monitored for disease symptoms for 7 days. (B) Percent survivals, (C) total percentage of diarrhea during the infection period, (D) daily percentage of diarrhea during the infection period, and (E) weight change of four groups of mice were plotted. Data are presented as mean ± SD (*n* = 10). ∗, *P* < 0.05, compare between group R20291 and group CUUG37785 + R20291. ns, no significantly.

We further evaluated whether or not the strain CCUG37785 can protect mice against infection with the strain R20291. The experimental scheme is illustrated in Fig. 7A. Four groups of mice (*n* = 10) were used. Group 1 (CCUG37785) was challenged with 10^6^ CCUG37785 spores as a control; Group 2 (R20291) was challenged with 10^6^ R20291 spores as a non-treatment group; Group 3 (CCUG37785/R20291) was given 10^6^ CCUG37785 spores, followed by challenge with 10^6^ R20291 spores; Group 4 (R20291/CCUG37785) was infected with 10^6^ R20291 spores, followed by administering 10^6^ CCUG37785 spores. Four groups of mice were monitored for mortality (Fig. 7B), weight loss (Fig. 7E) and diarrhea (Fig. 7C and D). While all mice in group R20291 developed diarrhea, only 20% mice from the group CCUG37785 + R20291 developed diarrhea (Fig. 7C and D). All mice in group CCUG37785 + R20291 mice survived, which is significantly higher than survival of group R20291 mice (60%) (*P < 0.05) (Fig. 7B). Mice in group R20291 also lost significantly more weight than those in group CCUG37785 + R20291 (*P* < 0.05, on postinfection days 2 and 3). As expected, mice from the control group CCUG37785 did not develop any signs of disease, neither diarrhea (Fig. 7C and D) nor weight loss (Fig. 7E), and all mice survived (Fig. 7B). In group R20291 + CCUG37785, 90% mice developed diarrhea (Fig. 7C and D) and 70% mice survived (Fig. 7B). Also, there was no significant difference between the weight loss of group R20291 + CCUG37785 and group R20291 (Fig. 7E). These results indicate that mice treated with CCUG37785 spores after infection with R20291 only provided limited protection against challenge with strain R20291.

### Pretreatment of mice with CCUG37785 spores before infection with R20291 spores decrease the R20291 spores and toxin levels in feces.

**FIG 8:**
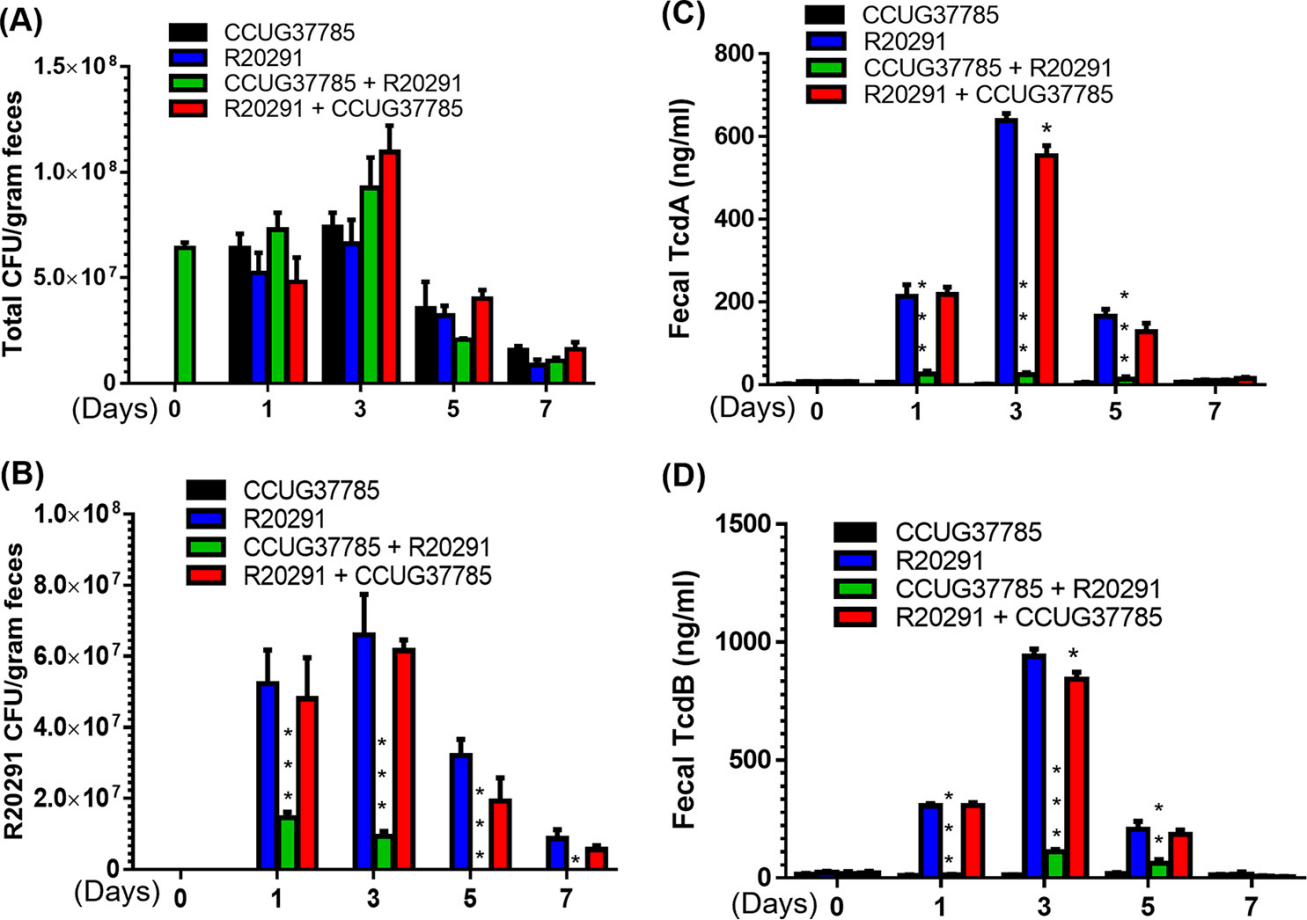
*C. difficile* spores and toxin levels in fecal samples of mice in four groups. (A) Total *C. difficile* spore numbers; (B) *C. difficile* R20291 spore numbers; (C) Tcd A level; (D) Tcd B level. Comparisons were made between groups (CCUG37785 + R20291) or (R20291 + CCUG37785) and R20291. (Experiments were repeated 3 times, and representative data were shown. Data are present as “Mean±SD”. ∗, *P* < 0.05; ∗∗, *P* < 0.01; ∗∗∗, *P* < 0.001).

Mice from the group CCUG37785 + R20291 excreted much less toxins in feces as compared with the group R20291 (Fig. 8C and D). No toxins were detected in feces from group CCUG37785 (Fig. 8C and D). However, group R20291 + CCUG37785 excreted less TcdA (Fig. 8C, *P < 0.05) and TcdB (Fig. 8D, *P < 0.05) than group R20291 only in day 3 and provided limited protection.

Without differentially enumerating CCUG37785 and R20291 spores from mouse feces, we could not determine R20291 spore changes in the gut (Fig. 8A). Therefore, we used PCR to assess the proportion of the R20291 in the C. difficile spores of fecal samples. As shown in Fig. 8B, pretreatment of mice with CCUG37785 before infection (group CCUG37785 + R20291) secreted 70% less amount of C. difficile R20291 spores on day 1, 90% less on day 3, and R20291 spores were not detected on days 5 and 7. Treatment with CCUG37785 spores after infection (group R20291 + CCUG37785), on the other hand, only slightly reduced R20291 spores from day 3 (*P* = 0.6710). Interestingly, in the group R20291 + CCUG37785, the proportion of the R20291 spores in fecal samples decreased over time; it is about 70% on day 3, 50% on day 5, and 40% on day 7, suggesting that CCUG37785 gradually replaced R20291 in the gut.

## DISCUSSION

In this study, we demonstrated that PaLoc and CdtLoc loci in strain CCUG37785 are replaced with a conserved 115-bp fragment and a conserved 68-bp fragment in these two regions, respectively, conforming to most NTCD strains. We phenotypically characterized strain CCUG37785 in comparison with C. difficile R20291 and found that CCUG37785 exhibited significantly higher adhesion and sporulation rates, similar motile ability, but lower spore germination rates and biofilm formation (Fig. 6). The ability to adhere to colonic mucosal cells or mucus is important for C. difficile to establish colonization (14, 17). Therefore, strain CCUG37785 could displace R20291 in the intestine for its significantly higher adhesion and sporulation rates, thereby potentially preventing recurrent CDI.

The data presented here demonstrate that strain CCUG37785 can readily colonize mice but does not cause pathology (Fig. 7). Further, strain CCUG37785 was able to adequately protect mice from subsequent pathogenic C. difficile R20291 infection (Fig. 7), but oral inoculation of CCUG37785 spores after R20291 infection only provided limited protection. These data provide insight into potential mechanisms of protection by strain CCUG37785 possible through colonization resistance. The first colonizing strain of one bacterial species metabolically outcompetes subsequent strains for space and resources (39, 40). In agreement, we found that the CCUG37785 prevention group (CCUG37785 + R20291) secreted much less R20291 spores (Fig. 8B), and much less TcdA and TcdB accordingly (Fig. 8C and D); While the CCUG37785 treatment group (R20291 + CCUG37785) secreted slightly less amount of R20291 spores (Fig. 8B), and slightly less TcdA and TcdB (Fig. 8C and D).

It is known that biofilm formation may confer bacterial cells including C. difficile higher resistance to antibiotics in comparison with planktonic cells. Interestingly, we found that strain CCUG37785 exhibited significantly lower biofilm formation capability than strain R20291, indicating that CCUG37785 could be more susceptible to antibiotic exposure. Indeed, we found that strain R20291 has higher values of MICs on several antibiotics tested (data not shown). However, very limited information is available on clinical effects of C. difficile biofilm formation *in vivo*.

Taken together, C. difficile CCUG37785 is safe and can be used as a therapeutic strain for the prevention of primary and recurrent CDI in mice; C. difficile CCUG37785 also has advantage of high sporulation rates and high adhesion capability to displace toxigenic strain to establish colonization. Our data is also in support of previous reports (14, 41, 42), which showed that intentional colonization of healthy subjects with NTCD was safe and that NTCD had no major adverse effects when given to healthy subjects with or without antibiotics (41).

In our previous study, we engineered the nontoxigenic strain CCUG37785 to express mTcd138 as a promising oral vaccine candidate against CDI (19). Data in our current study not only highlight the potential use of CCUG37785 to prevent primary and recurrent CDI in humans but also further support its use as an oral mucosal vaccine carrier against CDI. However, toxigenic strain 630Δerm was found to be able to transfer the PaLoc to NTCD strain CD37 *in vitro* (43), albeit with very low efficiency. Therefore, we should bear this possibility of conversion from NTCD to toxigenic strains in mind although no such conversions have been reported *in vivo* so far.

## MATERIALS AND METHODS

### Genome analysis of *C. difficile* CCUG37785 strain.

Total genomic DNA of the C. difficile CCUG37785 was extracted with a Qiagen genomic DNA extraction kit (Qiagen, USA). After confirming adequate quality of the extracted DNA using agarose gel electrophoresis, and nanodrop- and Qubit- (Thermo Fisher Scientific, USA) based quantification, whole genome sequencing was performed with Illumina technology at Beijing Novogene Bioinformatics Technology Co Ltd. The Illumina platform with massive parallel sequencing (MPS) was used and paired-end (500 bp insert) and mate-pair (5000 bp insert) libraries were constructed.

After assessing the quality of the sequencing reads using FastQC (44), the reads were *de novo* assembled into contigs using Qiagen CLC Genomics Workbench 11.0.1 (45) using the default settings. Assembled contigs were ordered against the C. difficile reference strain CD630 (GenBank accession no: CP010905.2) using Mauve (version 2.4.0) (46, 47). The draft genome of the CCUG37785 thus obtained was annotated using Rapid Annotation using Subsystems technology (RAST) web server (48). GLIMMER3 (49) was used to call protein-encoding genes, and the tRNAscan-SE tool (50) and “search_for_rnas” tool (developed by Niels Larson, unpublished) were used to call tRNA and rRNA encoding genes, respectively. Web-based Multi Locus Sequence Typing (MLST), using the seven housekeeping genes - adk, atpA, glyA, sodA, dxr, recA and tpi, was conducted with the public MLST tool maintained by the Center for Genomic Epidemiology (51). This Whole Genome Shotgun project has been deposited at DDBJ/ENA/GenBank under the accession JAGKRT000000000. The version described in this paper is version JAGKRT010000000.

### Phylogenetic analysis of nontoxigenic C. difficile strains.

Previously isolated and sequenced nontoxigenic C. difficile strains were identified in the literature. For each strain (Table 1), the appropriate genome sequence or accession number was sourced from GenBank. Additional information listed in Table 1 was also gathered from GenBank entries, and the number of plasmids was predicted using PlasmidFinder (20). The sequences and accession numbers were uploaded to the Type Strain Genome Server (TYGS) (25) which compared the genomes using the Genome BLAST Distance Phylogeny approach (GBDP) (52) and generated a minimum evolution tree.

### Searching for prophages.

Putative prophages were detected in the bacterial genome using PHASTER (53, 54), which screens for regions with high similarity to a database of phage genes. The software assigned prophage regions a score that determines whether they are considered complete (>90), questionable (70–90), or incomplete (<90).

### Annotation of putative prophage-like regions.

Putative prophage FASTAs were downloaded from PHASTER and submitted for automated annotation by the RAST tool kit (RASTtk) annotation engine, an updated version of the Rapid Annotations using Subsystems Technology (RAST) platform (48, 55, 56). The appropriate taxonomy I.D. for C. difficile (1496) was specified, and the “fix frameshift” function was selected. Default settings were used for all other parameters. The functional assignments of each gene were manually curated using the NCBI BLAST-protein tool (https://blast.ncbi.nlm.nih.gov/Blast.cgi).

### Genome comparison.

BLAST Ring Image Generator (BRIG) (57) was used to generate comparisons between nontoxigenic strains and the reference strain CD630. The upper, middle, and minimum identity percentage thresholds were set to 100%, 80%, and 50%, respectively.

### Analysis of toxin loci.

Sequences of nontoxigenic C. difficile were downloaded from GenBank and then the previously annotated pathogenicity locus (PaLoc) and surrounding genes of each strain were color-coded in Artemis. Color-coded annotations were imported into EasyFig software to generate BLAST comparisons. Upstream and downstream genes from PaLoc were identified in the genome annotations based on previous work describing the conserved *cdu* and *cdd* genes in these areas (31). The sequences between the cdu1 and cdd1 genes were extracted in Artemis and aligned using MAFFT (58) through the online MPI Bioinformatics Toolkit (59, 60). A similar method was used to analyze the binary toxin locus (*Cdt*) and surrounding genes. Genes associated with CdtLoc as well as conserved sequenced in nontoxigenic strains were identified using prior studies (5, 36).

### Animals.

All studies followed the Guide for the Care and Use of Laboratory Animals of the National Institutes of health and were approved by the Institutes Animal Care and Use Committee (IACUC) at University of South Florida. Wild-type C57BL/6 mice were purchased from Charles River Laboratories.

### Preparation of C. difficile spores.

Sporulation of the *C. difficile* R20291 and CCUG37785 strains were induced in Clospore medium as described previously (61). Briefly, an overnight 20 mL of *C. difficile* cultured in Columbia Broth was inoculated into 500 mL of Clospore medium and incubated for 1–2 weeks at 37°C in an anaerobic incubator. The spore culture was centrifuged at 10000g for 20 min, and the pellet was washed 5 times with sterile water and suspended in 10 mL of ddH_2_O. To purify the spores, 1-mL of spore suspension was layered onto the top of 10 mL of 50% (wt/vol) sucrose in water in a 15-mL tube (62). The gradient was centrifuged at 3200 × *g* for 20 min, and the spore pellet at the bottom was washed 5 times to remove the sucrose and was resuspended in sterile water. Spore preparations were > 99% pure, and spore concentration was determined by serial dilution on TCCA or BHI plates.

### Toxin expression assay.

To evaluate the toxin expression in C. difficile CCUG37785 and R20291, 10 mL of C. difficile cultures were collected at 12, 24, 36 and 48 h of post incubation. The cell density was adjusted to the same with fresh BHIS. The collected C. difficile cultures were centrifuged at 4°C, 8000 × *g* for 15 min, then the supernatants were filtered with 0.22 μm filter and used for ELISA. Anti-TcdA (PCG4.1, Novus Biologicals, USA) and anti-TcdB (AI, Gene Tex, USA) were used as coating antibodies for ELISA, HRP-Chicken anti-TcdA and HRP-Chicken anti-TcdB (Gallus Immunotech, USA) were used as detection antibodies.

### Sporulation and germination assay.

C. difficile sporulation and germination analysis were conducted as reported (63). *C. difficile* strains were cultured to mid-log phase in BHIS medium supplemented with 0.1% taurocholate (Sigma) at 37°C in an anaerobic chamber. A mixture of 70:30 sporulation medium (70% SMC medium and 30% BHIS medium containing 63 g Bacto peptone, 3.5g protease peptone, 11.1 g BHI medium, 1.5 g yeast extract, 1.06 g Tris base, 0.7 g NH_4_SO_4_, and 15 g agar per L) was prepared. Cultures were subsequently diluted to an optical density of 0.5 at OD_600_, then 150 μL of cultures were added and spread over the surface of a 70:30 medium agar plate. Approximately 24 h after the start of stationary phase (T_24_), C. difficile cells were scraped from the surface of the plate with a sterile inoculating loop and suspend in approximately 5 mL BHIS to an OD_600_ = approximately 1.0. 500 μl of samples from the sporulation medium were removed from the anaerobic chamber and mixed 1:1 with 95% ethanol for 15 min to kill vegetative cells. The samples were then returned to the anaerobic chamber, 100 μl of the ethanol-treated cultures were mixed with 100 μl of 10% taurocholate, and the mixtures were plated onto BHIS agar to induce C. difficile spore germination. The ethanol resistant CFU/mL was determined after incubation for 24h and was divided by the total CFU/mL of the nonethanol treated cultures.

For C. difficile germination analysis, the purified spores were diluted to an OD_600_ 0f 1.0 in the germination buffer (10 mM Tris (pH 7.5), 150 mM NaCl, 100 mM glycine, 10 mM taurocholic acid [TA]) to detect germination ratio. The value of OD_600_ was monitored immediately (0 min, t_0_), and was detected once every 2 min (t_x_) for 20 min at 37°C. The germination ratio was calculated as OD_600_ (Tx)/OD_600_ (T_0_). Spores in germination buffer without TA was used as the negative control.

### Adherence of C. difficile spores to HCT-8 cells.

The adherence of the *C. difficile s*pores to human gut epithelial cells was assessed as described previously (64). Briefly, HCT-8 cells were grown to 95% confluence (5 × 10^5^/well) in a 6-well plate and then moved into the anaerobic chamber, followed by infection with 5 × 10^6^ C. difficile spores at a multiplicity of infection (MOI) of 10:1. The plate was cultured at 37°C for 100 min in an anaerobic chamber. After incubation, the cell-spore mixture was washed three times with 1×PBS via centrifugation at 800 × *g* for 1 min to remove any unattached spores. The supernatants after centrifugation from each wash step were collected to enumerate any spores that did not adhere to the cells. The spores in the supernatant were enumerated on prereduced BHI agar supplemented with 10% taurocholic acid (wt/vol). The controls included PBS incubated with spores and RPMI incubated with spores, and the adhesion assays were performed in triplicate. The percentage of spore adherence was calculated using the following formula: (initial CFU/mL – eluted CFU/mL)/initial CFU/mL.

### Motility.

Motility assays were performed to assess the swimming and swarming behavior of the *C. difficile* strains (65, 66). C. difficile CCUG37785 and R20291 were cultured to optical OD_600_ of 0.8. For swimming analysis, 2 μl of different C. difficile cultures were penetrated into soft BHIS agar (0.175%) plates, meanwhile, 2 μl of cultures were dropped onto 0.3% BHIS agar plates for swarming analysis. The swimming assay plates were incubated for 24 h and the swarming plates were incubated for 48 h, respectively. Motility was quantitatively determined by measuring the radius of the zone of motility.

### Biofilm assay.

For biofilm analysis, CCUG37785 and R20291 were cultured to OD_600_ of 0.8, and 1% of C. difficile cultures were inoculated into Reinforced Clostridial Medium (RCM) with 8-well repeats in 96-well plate and incubated in the anaerobic chamber at 37°C for 48 h. The formation of biofilm was analyzed by crystal violet dye. Briefly, C. difficile cultures were removed with pipette carefully. Then 100 μl of 2.5% glutaraldehyde was added into the well to fix the bottom biofilm, and the plate was kept at room temperature for 30 min. Followed, the wells were washed with PBS for 3 times and dyed with 0.25% (wt/vol) crystal violet for 10 min. The crystal violet solution was disposed, and the wells were washed five times with PBS, followed by adding the acetone into wells to dissolve the crystal violet of cells. The dissolved solution was further diluted with ethanol for 2–4 times and then detected at OD_570_.

### Mouse model of C. difficile infection.

A mouse model of C. difficile infection was established as described previously (67, 68). Four groups of C57BL/6 mice (wild-type, *n* = 10) were given a mixture of five antibiotics including kanamycin (0.4 mg/mL), gentamicin (0.035 mg/mL), colistin (850 U/mL), metronidazole (0.215 mg/mL), and vancomycin (0.045 mg/mL) in the drinking water for 4 days. After 4 days of antibiotic treatment, all mice were given autoclaved water for 2 days, followed by a single dose of clindamycin (10 mg/kg) intraperitoneally (i.p.) 1 day before (day –1) challenge with 10^6^ C. difficile R20291 (for groups 2, 3 and 4) or CCUG37785 (for group 1) spores/mouse by gavage (day 0). Group 1 (CCUG37785) was challenged with 10^6^ C. difficile CCUG37785 spores on day 0; Group 2 (R20291) was challenged with 10^6^ C. difficile R20291 spores on day 0; Group 3 (CCUG37785+R20291) was given 10^6^ C. difficile CCUG37785 spores by gavage on days -1 and -2, followed by challenge with 10^6^ C. difficile R20291 spores on day 0; Group 4 (R20291+CCUG37785) was infected with 10^6^ C. difficile R20291 spores on day 0, followed by given 10^6^ C. difficile CCUG37785 spores by gavage on days 2 and 3. The animals were monitored daily for weight changes, diarrhea and survival, and moribund animals were euthanized. The fecal samples are collected on days 0, 1, 3, 5 and 7 postchallenge.

### Evaluation of C. difficile spore numbers and toxin levels in feces.

Fecal samples were collected on postinfection days 0, 1, 3, 5 and 7, and stored in –20°C freezer. Prior to use, 50 mg of feces were dissolved with 500 μl PBS (0.1 g/mL) and treated with 500 μl of absolute ethanol (Sigma-Aldrich) for 60 min at room temperature. Then, samples were thoroughly suspended, serially diluted, and plated onto selective medium supplemented with taurocholate (0.1% wt/vol), Cefoxitin (16 μg/mL), d-cycloserine (250 μg/mL) (TCCA plates). The plates were incubated anaerobically at 37°C for 48 h before the colonies were counted, and the results were expressed in CFU/gram. To determine the toxin levels in feces, 0.1 g/mL of fecal solutions containing protease inhibitor cocktail were diluted 2-fold with PBS and used for determination of TcdA and TcdB levels by ELISA.

To determine the proportion of R20291 spores in the C. difficile spores of fecal samples, colony PCR was performed to amplify the *tcdB* gene in R20291 to distinguish R20291 from NTCD. Quick C. difficile genomic DNA extraction was conducted as reported earlier (69). Briefly, a C. difficile colony was suspended in a microcentrifuge tube containing 1× PCR buffer with fresh lysozyme solution (500 μg/mL final concentration), incubate 15 min at room temperature. Then, proteinase K (200 μg/mL final concentration) was added and incubated for 1 h at 58°C, followed by heating for 15 min at 90°C. Crude genomic extract was used for PCR using primers: TcdB-F (GTATTACCTAATGCTCCAA) and TcdB-R (CACCTTCATAGTTATCTCTT).

### Statistical analysis.

Data were analyzed by Kaplan-Meier survival analysis with a log rank test of significance, by analysis of variance (ANOVA), and by one-way or two-way ANOVA followed with Bonferroni post-tests using the Prism statistical software program. Results are expressed as means ± standard errors of means. Differences were considered statistically significant if *P* < 0.05.

### Data availability.

The data that support the findings of this study are available from the corresponding author, X.S., upon reasonable request. The Whole Genome Shotgun project for the nontoxigenic C. difficile strain CCUG37785 has been deposited at DDBJ/ENA/GenBank under the accession JAGKRT000000000. The version described in this paper is version JAGKRT010000000.
