# Supplementary material for: Genomic and Phenotypic Characterization of the Nontoxigenic Clostridioides difficile Strain CCUG37785 and Demonstration of Its Therapeutic Potential for the Prevention of C. difficile Infection
Source: Microbiol Spectr. 2022 Mar 22;10(2):e01788-21. doi: 10.1128/spectrum.01788-21 (PMC9045287; doi:10.1128/spectrum.01788-21)
Supplement: SUPPLEMENTAL FILE 1 — Supplemental material. Download SPECTRUM01788-21_Supp_1_seq12.pdf, PDF file, 0.2 MB [file spectrum01788-21_supp_1_seq12.pdf]

Supplementary Information:

Genomic and phenotypic characterization of the nontoxigenic *Clostridioides difficile* strain CCUG37785 and demonstration of its therapeutic potential for the prevention of *C. difficile* infection

Shaohui Wang[a]#, Joshua Heuler[a]#, Ishani Wickramage[a], Xingmin Sun[a]*

[a]*Department of Molecular Medicine, Morsani College of Medicine, University of South Florida, Tampa, FL, United States.*

# Equal contribution

* Corresponding author (sun5@usf.edu).

Supplementary figures and figure legends

Supplementary Fig. 1. Toxin expression assay. Strain R20291 produced both TcdA and TcdB, with the highest toxin concentrations of TcdA (A) and TcdB (B) detected at 48 hours post-inoculation; Strain CCUG37785 produced neither TcdA (A) nor TcdB (B).

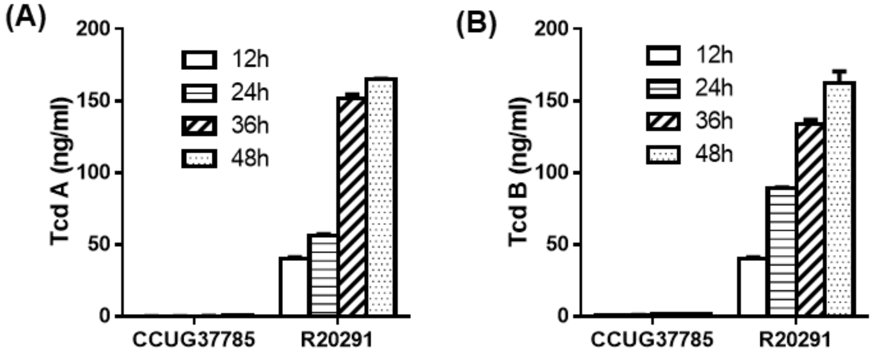

Supplementary tables

Supplemenary Table 1. BLASTn comparisons between CCUG37785 and other non-toxigenic strains. The nucleotide sequences of non-toxigenic strains in Table 1 were used as queries against CCUG37785. Searches were performed online through NCBI (https://blast.ncbi.nlm.nih.gov/Blast.cgi).

Strain	Identity (%)	Query Cover (%)	E-value
DSM28666	99.47	95	0
DSM28669	99.72	94	0
DSM28670	98.43	92	0
DSM29629	98.41	91	0
DSM29637	98.42	94	0
DSM29688	99.69	93	0
Z31	99.54	99	0

Supplementary Table 2. CCUG37785 prophage region 3 draft annotation. Prophage regions predicted by PHASTER were annotated using the RASTtk automated pipeline followed by curating putative protein functions using NCBI BLASTp searches. All matches displayed at least 80% sequence identity with the query amino acid sequence.

ORF	Start	Stop	Strand	Putative Function	E-value
1	1335	205	-	site-specific integrase	0
2	2081	1575	-	ImmA/IrrE family metallo-endopeptidase	2.00E-114
3	2705	2157	-	helix-turn-helix domain-containing protein	4.00E-124
4	3058	2717	-	XRE family transcriptional regulator	1.00E-77
5	3235	3438	+	helix-turn-helix domain-containing protein	6.00E-39
6	3486	4295	+	phage antirepressor	0
7	4342	4551	+	hypothetical protein	6.00E-41
8	4935	4561	-	DUF6173 family protein	2.00E-85
9	5011	5208	+	helix-turn-helix transcriptional regulator	7.00E-36
10	5239	5631	+	helix-turn-helix domain-containing protein	5.00E-85
11	5618	5761	+	hypothetical protein	2.00E-20
12	5832	6257	+	hypothetical protein	5.00E-89
13	6269	7435	+	DUF2800 domain-containing protein	0
14	7450	7785	+	hypothetical protein	4.00E-71
15	7838	8410	+	DNA-directed RNA polymerase sigma-70 factor	4.00E-135
16	8433	9005	+	DUF2815 family protein	1.00E-36
17	9005	10963	+	DNA polymerase	0
18	10975	13392	+	virulence-associated E family protein	0
19	13674	14000	+	VRR-NUC domain-containing protein	3.00E-70
20	14030	15319	+	DEAD/DEAH box helicase family protein	0
21	15343	15480	+	hypothetical protein	9.00E-32
22	15473	15952	+	helix-turn-helix domain-containing protein	3.00E-107
23	16091	16348	+	hypothetical protein	1.00E-51
24	16536	17579	+	AAA family ATPase	0
25	17580	19802	+	S8 family peptidase	0
26	19960	20643	+	phage terminase small subunit	7.00E-164
27	20633	21874	+	PBSX family phage terminase large subunit	0
28	21880	23319	+	portal	0
29	23309	24802	+	portal	0
30	24768	25010	+	minor capsid	0
31	25320	25883	+	hypothetical protein	6.00E-48
32	25895	26785	+	phage scaffolding protein	6.00E-131
33	26804	27037	+	capsid protein	0
34	27047	27448	+	hypothetical protein	4.00E-90
35	27442	27789	+	hypothetical protein	3.00E-77
36	27789	28214	+	HK97 gp10 family phage protein	3.00E-100
37	28207	28644	+	hypothetical protein	2.00E-99
38	28637	28813	+	hypothetical protein	8.00E-32
39	28814	30124	+	phage tail sheath family protein	0

Continued.

Table 2 Cont.

ORF	Start	Stop	Strand	Putative Function	E-value
40	30141	30611	+	phage tail tube protein	2.00E-111
41	30683	31123	+	phage portal protein	2.00E-102
42	31198	31317	+	hypothetical protein	8.00E-16
43	31966	32070	+	hypothetical protein	2.00E-14
	32363	32391	+	CRISPR repeat with sequence gttttagattaactatatggaatgtaaat	
	32363	32810	+	CRISPR region with repeat gttttagattaactatatggaatgtaaat	
	32392	32428	+	CRISPR spacer	
	32429	32457	+	CRISPR repeat with sequence gttttagattaactatatggaatgtaaat	
	32456	32638	+	hypothetical protein	
	32458	32495	+	CRISPR spacer	
	32496	32524	+	CRISPR repeat with sequence gttttagattaactatatggaatgtaaat	
	32525	32561	+	CRISPR spacer	
	32562	32590	+	CRISPR repeat with sequence gttttagattaactatatggaatgtaagt	
	32591	32626	+	CRISPR spacer	
	32627	32655	+	CRISPR repeat with sequence gttttagattgactatatggaatgtaaat	
	32656	32693	+	CRISPR spacer	
	32694	33400	+	CRISPR region with repeat gttttagattaactatatggaatgtaaat	
	32694	32722	+	CRISPR repeat with sequence gttttagattaactatatggaatgtaaat	
	32723	32781	+	CRISPR spacer	
	32782	32810	+	CRISPR repeat with sequence gttttagattaactatatggaatgtaaat	
	32811	32846	+	CRISPR spacer	
	32847	32875	+	CRISPR repeat with sequence gttttagattaactatatggaatgtaaat	
	32876	32912	+	CRISPR spacer	
	32913	32941	+	CRISPR repeat with sequence gttttagattaactatatggaatgtaaat	
	32942	32975	+	CRISPR spacer	
	32976	33004	+	CRISPR repeat with sequence gttttagattaactatatggaatgtaaat	
	33005	33040	+	CRISPR spacer	
	33041	33069	+	CRISPR repeat with sequence gttttagattaactatatggaatgtaaat	
	33070	33107	+	CRISPR spacer	
	33108	33136	+	CRISPR repeat with sequence gttttagattaactatatggaatgtaaat	
	33137	33173	+	CRISPR spacer	
	33174	33202	+	CRISPR repeat with sequence gttttatattaactatgtggtatgtaaat	
	33203	33239	+	CRISPR spacer	
	33240	33268	+	CRISPR repeat with sequence gttttatattaactatgtggtatgtaaat	
	33269	33304	+	CRISPR spacer	
	33305	33333	+	CRISPR repeat with sequence gttttagattaactatatggaatgtaaat	
	33334	33371	+	CRISPR spacer	
	33372	33400	+	CRISPR repeat with sequence gttttagattaactatgtggagaaaaact	
44	33560	33730	+	ribbon-helix-helix domain-containing protein	5.00E-32
45	33858	34700	+	phage repressor	0
46	34755	34910	+	hypothetical protein	5.00E-21
47	35091	34954	-	hypothetical protein	9.00E-23
48	35467	36237	+	SHOCT domain-containing protein	0
49	36295	38646	+	tape measure protein	0
50	38663	39358	+	LysM peptidoglycan-binding domain-containing protein	7.00E-168

Continued.

Table 2 Cont.

ORF	Start	Stop	Strand	Putative Function	E-value
51	39351	41243	+	NlpC/P60 family protein	0
52	41257	41517	+	DUF2577 domain-containing protein	9.00E-54
53	41522	41941	+	DUF2634 domain-containing protein	8.00E-96
54	41942	42991	+	baseplate J/gp47 family protein	0
55	42984	43601	+	YmfQ family protein	9.00E-148
56	43613	44638	+	phage tail protein	0
57	44655	46196	+	hypothetical protein	0
58	46210	46503	+	hypothetical protein	1.00E-61
59	46503	46685	+	hypothetical protein	4.00E-34
60	46719	46910	+	hypothetical protein	3.00E-35
61	46924	47556	+	tail fiber	2.00E-148
62	47567	47770	+	hypothetical protein	2.00E-35
63	48025	47795	-	no significant similarity found	n/a
64	48062	48667	+	tail fiber protein	1.00E-38
65	48691	48996	+	hypothetical protein	3.00E-61
66	48996	49160	+	hypothetical protein	1.00E-28
67	49360	50709	+	hypothetical protein	0
68	50709	50888	+	hypothetical protein	5.00E-33
69	50929	51159	+	hemolysin XhlA family protein	2.00E-45
70	51179	51436	+	phage holin family protein	2.00E-52
71	51697	52023	+	abi-like family protein	5.00E-70
72	52063	52560	+	abi-like family protein	3.00E-15
73	52639	53451	+	N-acetylmuramoyl-L-alanine amidase	0
74	53738	55747	+	cell wall-binding repeat-containing protein	0
75	55977	55792	-	AbrB/MazE/SpoVT family DNA-binding domain-containing protein	9.00E-34
76	56334	56206	-	putative membrane protein	9.00E-17
